# Mutation-related differences in exploratory, spatial, and depressive-like behavior in *pcd* and *Lurcher* cerebellar mutant mice

**DOI:** 10.3389/fnbeh.2015.00116

**Published:** 2015-05-12

**Authors:** Jan Tuma, Yaroslav Kolinko, Frantisek Vozeh, Jan Cendelin

**Affiliations:** ^1^Laboratory of Neurodegenerative Disorders, Faculty of Medicine in Pilsen, Biomedical Centre, Charles University in PraguePilsen, Czech Republic; ^2^Department of Pathophysiology, Faculty of Medicine in Pilsen, Charles University in PraguePilsen, Czech Republic; ^3^Department of Histology and Embryology, Faculty of Medicine in Pilsen, Charles University in PraguePilsen, Czech Republic

**Keywords:** *Lurcher*, olivocerebellar degeneration, *pcd*, spatial learning, water maze

## Abstract

The cerebellum is not only essential for motor coordination but is also involved in cognitive and affective processes. These functions of the cerebellum and mechanisms of their disorders in cerebellar injury are not completely understood. There is a wide spectrum of cerebellar mutant mice which are used as models of hereditary cerebellar degenerations. Nevertheless, they differ in pathogenesis of manifestation of the particular mutation and also in the strain background. The aim of this work was to compare spatial navigation, learning, and memory in *pcd* and *Lurcher* mice, two of the most frequently used cerebellar mutants. The mice were tested in the open field for exploration behavior, in the Morris water maze with visible as well as reversal hidden platform tasks and in the forced swimming test for motivation assessment. *Lurcher* mice showed different space exploration activity in the open field and a lower tendency to depressive-like behavior in the forced swimming test compared with *pcd* mice. Severe deficit of spatial navigation was shown in both cerebellar mutants. However, the overall performance of *Lurcher* mice was better than that of *pcd* mutants. *Lurcher* mice showed the ability of visual guidance despite difficulties with the direct swim toward a goal. In the probe trial test, *Lurche*r mice preferred the visible platform rather than the more recent localization of the hidden goal.

## Introduction

Neurodegenerative disorders affecting the olivo-cerebellar system are manifested by well-characterized motor disorders. Nevertheless, the cerebellum is also involved in cognitive and behavioral processes, abnormalities of which have been described in humans (Schmahmann and Sherman, 1997; Cooper et al., 2010; Fancellu et al., 2013; Marien and Beaton, 2014), as well as in a wide spectrum of cerebellar mutant mice (for review see Manto and Marmolino, 2009; Cendelin, 2014). Cerebellar mutants are variable relative to the feature and extent of the cerebellar and extra-cerebellar neuronal degeneration. Moreover, the mutations appear in different mouse strains and these mutants retain specific phenotypic traits of the original strains. The importance of the genetic background for behavioral manifestation has been shown in gain-of-function (Cendelin et al., 2014) as well as loss-of-function mutations (Lalouette et al., 2001). Furthermore, the review by D'hooge and de Deyn (2001) showed that sex differences, age, nutrition, stress, infections as well as experimental protocol, apparatus, and data analysis could markedly influence results in the Morris water maze task (Morris, 1984). With respect to these facts, it is therefore difficult to compare the behavioral phenotype of various mutations in mice of different background strains from different studies. On the other hand, the identification and understanding of specific impairments related to a particular mutation should be of interest regarding the variability of human hereditary cerebellar degenerations (Manto, 2005) and the use of mouse models for the development of disease-targeted therapeutic approaches.

In the present study, the behavioral phenotype of two of the most frequently used mouse models of olivocerebellar degeneration, *Lurcher* and *Purkinje cell degeneration* (*pcd*), were studied. *Lurcher* mice (Phillips, 1960) constitute the semi-dominant gain-of-function mutation in the δ2 glutamate receptor (GluRδ2) gene that changes the receptor into a leaky membrane channel, which chronically depolarizes the cell membrane (Zuo et al., 1997). GluRδ2 is expressed predominantly by Purkinje cells (Araki et al., 1993) and therefore, cell-autonomous degeneration of Purkinje cells is a primary effect of the mutation (Wetts and Herrup, 1982a,b). Virtually all Purkinje cells disappear by 3 months of age (Caddy and Biscoe, 1979). Fast reductions of cerebellar interneurons and inferior olive neuron numbers are due to secondary target-related cell death (Caddy and Biscoe, 1979; Wetts and Herrup, 1982a,b; Zanjani et al., 2006). *Lurchers* are characterized by ataxia (Fortier et al., 1987), spatial orientation impairments (Lalonde et al., 1988; Cendelin et al., 2008), and alterations of anxiety-related behaviors (Hilber et al., 2004). *Lurcher* mutation exists in two phenotypically undistinguishable alleles, the original one, *Grid2^Lc^* (Zuo et al., 1997), and *Grid2^Lc−J^* (de Jager et al., 1997). For experiments, *Grid2^Lc^* mutants have been used, e.g., in B6CBA and C3H (Caddy and Biscoe, 1979; Cendelin et al., 2014) strain backgrounds.

*Pcd* mice (Mullen et al., 1976) carry a recessive loss-of-function mutation in the gene encoding the cytosolic ATP/GTP binding protein 1 (*Agtpb1*), a.k.a. *Nna1* (Fernandez-Gonzalez et al., 2002). *Nna1* is expressed throughout the brain and retina with prominence in cerebellar Purkinje cells (Mullen et al., 1976; Baltanas et al., 2011), mitral cells of the olfactory bulb (Greer and Shepherd, 1982), thalamic neurons (O'Gorman, 1985; O'Gorman and Sidman, 1985), and retinal photoreceptors (Blanks et al., 1982; Lavail et al., 1982). Histopathological analysis of *pcd* mice revealed rapid Purkinje cell loss between the third and fourth postnatal week (Baltanas et al., 2013), slowly progressive cerebellar granule cell degeneration, moderate reduction of the deep cerebellar nuclei, and slow degeneration of inferior olivary neurons that are supposed to be secondary to the loss of Purkinje cells (Ghetti et al., 1987; Triarhou et al., 1987). The photoreceptor decrease progresses slowly and even after 9 months of life, some photoreceptors are retained (Marchena et al., 2011). *Pcd* mice suffer from ataxia (Mullen et al., 1976; Goodlett et al., 1992) and a deterioration of spatial navigation learning (Goodlett et al., 1992). The *pcd* mutation exists in several different alleles (Wang and Morgan, 2007). *Pcd* mice carrying the original allele *Agtpbp1^pcd^* have been used for experiments, e.g., in B6.BR (Vinueza Veloz et al., 2014), C57BL/6J (Zhang et al., 1996), or B6C3Fe (Rotter et al., 2000) strains.

Both mutants constitute a distinct type of mutation affecting the olivo-cerebellar system either exclusively (*Lurcher*) or inclusively (*pcd*) and determining a strong pathological phenotype. Distinct histopathological similarities predestine them to frequent mutual comparisons, mostly often indirect (Furuya et al., 1994; Lalonde and Thifault, 1994; Le Marec and Lalonde, 1997, 2000), but none of these studies have involved systematic experiments. Therefore, the aim of this study was to test the behavioral phenotype of *pcd* and *Lurcher* mice with particular attention paid to cognitive and emotional disturbances under the same environmental conditions. We also aimed to assess the comparability of the mutants, which are not commercially available in identical strains. Thus, healthy littermates were also tested to assess the role of the genetic backgrounds.

## Materials and methods

### Animals

Two cohorts of adult (3 months) B6.BR *pcd^1J^* and B6CBA *Lurcher* mutants and their healthy wild type littermates of both sexes were used (for *n*, see Table 1). Both B6.BR *pcd* and wild type mice were obtained by crossing heterozygous males and females. Both B6CBA *Lurcher* and wild type mice were obtained by crossing wild type females with heterozygous *Lurcher* males. All animals were housed in the same breeding facility under standard laboratory conditions in a temperature and humidity controlled room with a 12/12 h light/dark cycle (6 a.m. to 6 p.m.). The tests were performed during the light phase of the cycle. Animals were kept in plastic cages with wooden shavings and maintained with a standard commercial pellet diet and water *ad libitum*. All experimental procedures were performed in compliance with the EU Guidelines for Scientific Experimentation on Animals and with the permission of the Ethical Commission of the Faculty of Medicine in Pilsen.

**Table 1:** **Mean ± SEM (*n*) body weight of 3-month-old *pcd* and *Lurcher* mice and their healthy littermate controls (separately for both cohorts)**.

	**B6.BR**		**B6CBA**	
	***pcd***	**wild type**	***Lurcher***	**wild type**
**COHORT 1**				
Females (g)	15.96±1.262 (16)	21.57±0.986 (22)	20.49±1.847 (17)	22.00±1.887 (21)
Males (g)	19.56±2.350 (16)	26.51±2.509 (17)	24.79±1.847 (19)	27.89±1.885 (18)
**COHORT 2**				
Females (g)	15.53±2.911 (14)	22.32±1.052 (13)	20.01±1.507 (14)	22.92±1.168 (12)
Males (g)	19.03±3.199 (11)	27.81±1.519 (16)	24.56±1.272 (12)	28.47±1.574 (15)

### Experimental design

To eliminate the influence of the tests on behavior, two cohorts of mice were used. Cohort A was used for analysis of the explorative behavior in the open field and spatial learning, orientation and navigation in the Morris water maze. Cohort B was used for assessment of motivation and depressive-like behavior in the water environment. The body weight of mice from both cohorts was measured on the first day of the experiment before the tests. For behavioral tests and body weight evaluation, male and female mice were considered separate experimental groups. Since *pcd* mice are known to suffer from retinal degeneration (Blanks et al., 1982; Lavail et al., 1982), the retinas of samples of *pcd* mutants were examined stereologically and compared with those from their B6.BR wild type littermates as well as with retinas of B6CBA *Lurcher* and wild type mice to assess the presence and extent of photoreceptor degeneration at the time of finishing the behavioral testing.

### Behavioral testing

#### Open field

Explorative behavior and spontaneous motor activity were analyzed using the open field test. The apparatus consisted of a white open top plastic box (50 × 50 × 50 cm) with an illumination intensity of 20 lux at the box floor level. The subject was placed in the center of the open field and left undisturbed for 5 min. The apparatus was cleaned with 70% ethanol between subjects. The activity was recorded using EthoVision® XT 7.1 (Noldus Information Technology b.v., Netherlands). The locomotion activity (% of the test duration), distance moved (cm), thigmotaxis (% moved distance in the 3 cm border zone), and mean walking speed (cm/s) were evaluated.

#### Morris water maze task

The goal-directed navigation and spatial learning were evaluated using a Morris water maze task (Morris, 1984). The apparatus consisted of a circular white plastic pool (100 cm in diameter × 55 cm in height), with the water level set at a height of 35 cm above the base. The pool was filled with water (26 ± 2°C) and illuminated with 70 lux at the water surface. Escape from the water was provided by a transparent circular PlexiGlass platform (7.5 cm in diameter; 0.5 cm below the water level). Four starting points around the circumference of the pool were arbitrarily designated: North (N), South (S), West (W), and East (E). Each animal performed four trials per day-session with 16 min inter-trial intervals. The subject was introduced into the pool facing the wall in one of four starting positions. The maximal time for the platform location was 60 s. If the mouse did not locate the platform within the allotted time, it was manually placed on the platform. After each trial, the mouse was left on the platform for 30 s.

**Figure 1:**
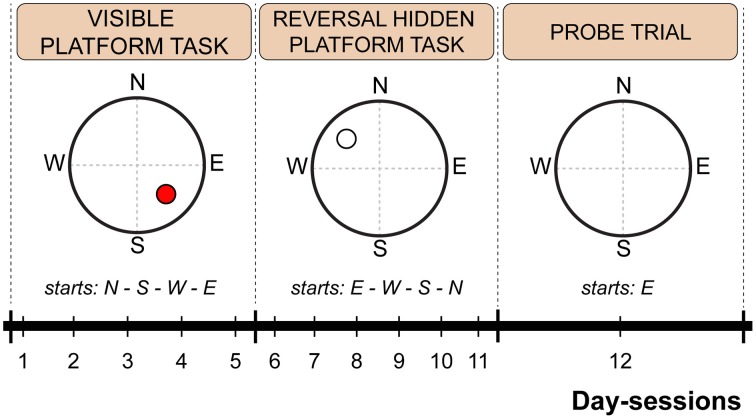
**Scheme of the Morris water maze protocol indicating time schedule, platform position and starting point sequence**. The red circle indicates a visible platform position. An empty circle indicates a hidden platform position. Dashed lines represent imaginary quadrant borders.

The water maze test consisted of 12 consecutive day-sessions arranged into three phases: visible platform test (day-sessions 1–5), reversal hidden platform test (day-sessions 6–11), and probe trial (day-session 12). For the visible platform test, the hidden escape platform position was highlighted by a cylindrical label (3 cm in diameter; 5 cm in height) with vertical black and white stripes mounted 12 cm above the submerged platform. The label served as a cue for visual goal-directed navigation. Platform position and starting point order is schematically depicted in Figure 1. For the probe trial, the escape platform was removed, and each mouse was allowed to swim freely for 60 s per trial.

The movement of the mice in the maze was recorded using EthoVision® XT 7.1. Escape latencies (s) and distance moved (cm) were measured as the basic parameters of the performance in the Morris water maze task. Swimming speed during periods of activity (i.e., excluding floating periods) was calculated to assess the swimming ability of the mice and to evaluate the relationship between latency and distance moved. Mouse navigation and orientation relative to the escape platform position was determined as the heading angle error and direct swim percentage. The heading angle error was measured as a deviation from a direct line from the starting point to the center of the platform. As direct swim, those trials with a shorter distance moved than the length of a direct line connecting the starting point and the platform multiplied by 1.3 were considered (Cendelin et al., 2014). The exploration strategy was evaluated using thigmotaxis (% moved distance in the 10 cm margin zone of the maze). Floating (% of time spent inactive) was assessed as a specific behavioral event. Spatial learning and memory were assessed using the preference for the NW or SW quadrant, respectively (% of distance moved), for the first 30 s of the first start of the probe trial only to avoid the effect of adaptation on the missing platform.

#### Forced swimming test

The motivation to swim and depressive-like behavior were analyzed using the Porsolt's forced swimming test (Porsolt et al., 1979). Mice were immersed in a glass water tank (diameter: 18 cm; height: 26 cm; water depth: 19 cm). The water was maintained at 26 ± 2°C and illuminated with 70 lux at the water surface. The mouse was left to swim without any possibility of escape for 15 min per day-session for three consecutive days. Immobility periods were recorded using EthoVision® XT 7.1, and relative immobility (% of total time) was calculated. To assess the development of depressive-like behavior within a day-session, immobility periods were evaluated separately in three 5-min time-bouts for each day-session.

### Quantitative histology of the retina

The presence and extent of retinal degeneration in *pcd* mutants (*n* = 8) compared with their healthy littermates (*n* = 8) and B6CBA mice (*Lurcher*: *n* = 8; WT: *n* = 8) was assessed using stereological analysis. Paraformaldehyde-fixed right eyes of four females and four males per group were processed into 10 μm thick serial sections with random orientation. Every fifteenth section was stained with Gill's hematoxylin and scanned as a stack of four 2.5 μm optical sections using an Olympus C-5060 digital camera coupled to an Olympus CX31 microscope (Olympus, Tokyo, Japan) using an 60× objective with a numerical aperture of 1.35. To count the retinal photoreceptor cell nuclei, nine dissector-counting frames were randomly imposed on each stack (Glaser et al., 2007), taking into account only those optical dissectors located in the outer nuclear layer (ONL) of the retina (352 ± 15 dissectors for each animal). The volume of the retina and total number of photoreceptor nuclei were estimated using the fractionator method. Finally, the number of photoreceptor nuclei was related to the retina volume and numerical density was determined (Gundersen, 1986; Boyce et al., 2010). The mean coefficient of sampling error (CE) was 4.7% for the ONL volume and 4.4% for the retina volume (Gundersen and Jensen, 1987).

### Statistical analysis

Data were analyzed using traditional statistical tests extended with a non-parametric permutational approach (Pesarin and Salmaso, 2010). Three-Way ANOVA or Three-Way ANOVA with repeated measurements were evaluated, and the following factors were analyzed: type—cerebellar mutant (CM)/wild type (WT), strain—B6CBA/B6.BR, sex—female/male, and within-group factors day-session and/or time-bout (session, bout; if applicable). Interactions of these factors were also assessed. All ANOVA-tests were followed by planned comparisons performed using *t*-tests with a Bonferroni correction for repeated measurements (day-session and/or time-bout). The data ordered in a paired design were analyzed using the paired *t*-test. The preference for the selected quadrants was verified using the one-sample *t*-test against a value of 25%, which represents a random occurrence. The data are presented as mean ± SEM. *p* < 0.05 was considered statistically significant. Reported *F* and *t*-values are considered as *F*_0_ and *t*_0_, respectively, before the start of permutational tests. ANOVAs and *t*-tests were performed with maximal 5000 and 10,000 permutations, respectively. Statistical analyses were conducted using the R version 3.1.2 for Mac OS.

## Results

### Body weight

The mean body weight of mice is presented in Table 1. Both *pcd* and *Lurcher* mutants showed significantly reduced body weights compared to their healthy counterparts. Moreover, *pcd* mice showed significantly lower body weights compared to *Lurchers*, even though the wild type mice for both groups were not different (for statistics see Supplementary Table 1).

### Open field

**Figure 2:**
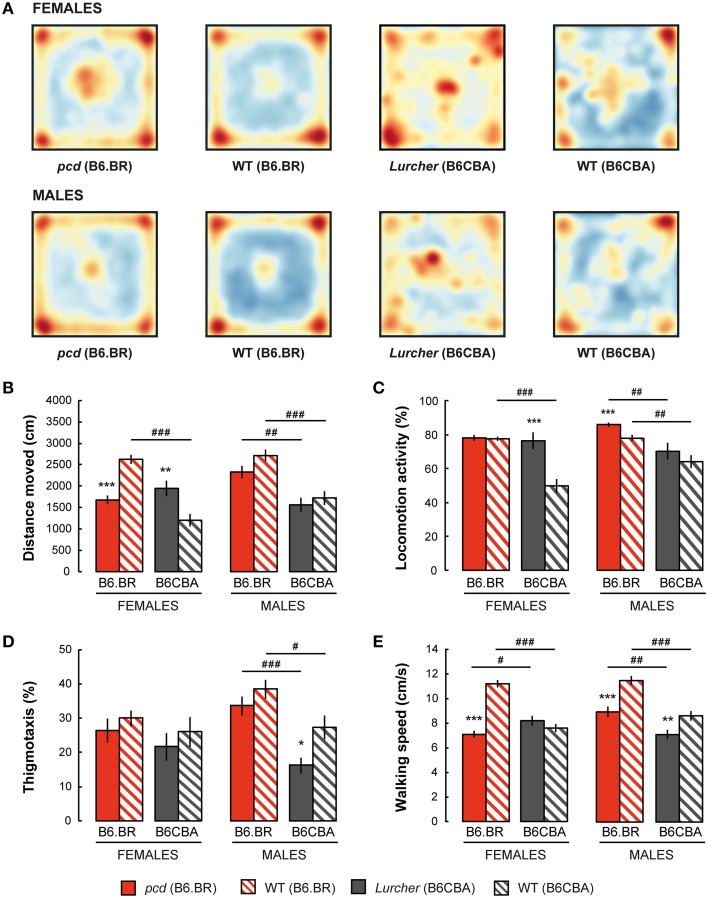
**Open field test: (A) Superposition of trajectories (frequency of animal presence) for females and males in the arena; (B) Total distance moved (cm); (C) Percentage of time spent with locomotion activity (%); (D) Percentage of thigmotaxis (% moved distance in the 3 cm border zone) and (E) Mean walking speed (cm/s) during locomotion activity periods**. Statistical significance was evaluated using a permutational *t*-test. Within-strain comparison: ^*^p < 0.05, ^**^p < 0.01, and ^***^p < 0.001. Between-strain comparison: ^#^ *p* < 0.05, ^##^ *p* < 0.01, and ^###^ *p* < 0.001. Data are presented as mean ± SEM.

Spatial distribution of the exploratory activity in the open field is presented in Figure 2A. Despite an evident preference for corners of the square arena in all experimental groups, B6CBA mice, and especially B6CBA *Lurchers*, showed a higher tendency to explore the entire arena. The significance of individual parameters measured in the open field on individual factors (type, strain, sex) and their interactions are shown in Table 2.

**Table 2:** **Open field test: statistical significances of the between-group factors (type, strain, and sex) and their interactions**.

**Between-group factors**	**Distance**		**Locomotion**		**Thigmotaxis**		**Walking speed**	
	***F*_(1, 138)_**	***p***	***F*_(1, 138)_**	***p***	***F*_(1, 138)_**	***p***	***F*_(1, 138)_**	***P***
Type	3.24	0.045	18.12	<0.001	6.32	0.007	55.21	<0.001
Strain	48.45	<0.001	37.04	<0.001	15.63	<0.001	49.22	<0.001
Sex	4.14	n.s.	2.80	n.s.	1.48	n.s.	4.28	0.020
Type:Strain	21.06	<0.001	6.12	0.005	0.54	n.s.	31.48	<0.001
Type:Sex	0.66	n.s.	1.76	n.s.	0.70	n.s.	0.30	n.s.
Strain:Sex	2.08	n.s.	0.00	n.s.	4.41	0.032	4.77	0.023
Type:Strain:Sex	12.46	<0.001	8.38	0.004	0.33	n.s.	13.01	<0.001

*Permutational Three-Way ANOVA*.

Distance moved is shown in Figure 2B. While *pcd* females moved a shorter distance in the open field than B6.BR wild type females, *Lurcher* females walked longer distances than B6CBA wild type females. In males, no significant differences were found between mutant and wild type mice. Wild type females and *pcd* and wild type males of the B6.BR strain had longer distances moved than did their B6CBA counterparts. *Pcd* males moved longer distances than *pcd* females (*t* = −3.68, *p* < 0.001) and B6CBA wild type males moved longer distances than females (*t* = −2.40, *p* < 0.020).

Locomotion activity is shown in Figure 2C. The activity was higher in *Lurcher* females than in B6CBA wild type females and in *pcd* males than in B6.BR wild type males. Strain comparison showed higher activity in B6.BR wild type females, mutant and wild type males than in their B6CBA counterparts. *Pcd* males had higher locomotion activity than *pcd* females (*t* = −3.76, *p* < 0.001), and B6CBA wild type males were more active than females (*t* = −2.52, *p* < 0.013).

Thigmotaxis is displayed in Figure 2D. Thigmotaxis was significantly lower in *Lurcher* males than in B6CBA wild type males. B6.BR males showed higher thigmotaxis than B6CBA males. There was no effect of sex on thigmotaxis in the open field.

The parameter walking speed is shown in Figure 2E. Walking speed in the open field arena was lower in *pcd* females than in B6.BR wild type females, in *pcd* males than in B6.BR wild type males, and in *Lurcher* males than in B6CBA wild type males. Strain comparison showed that *pcd* females walked slower than *Lurcher* females, but *pcd* males were faster than *Lurcher* males. Both B6.BR wild type females and males achieved a higher walking speed than B6CBA wild type mice. *Pcd* males showed higher walking speed than *pcd* females (*t* = −3.59, *p* < 0.001), and B6CBA wild type males walked faster than females (*t* = −2.02, *p* < 0.048).

### Morris water maze

**Figure 3:**
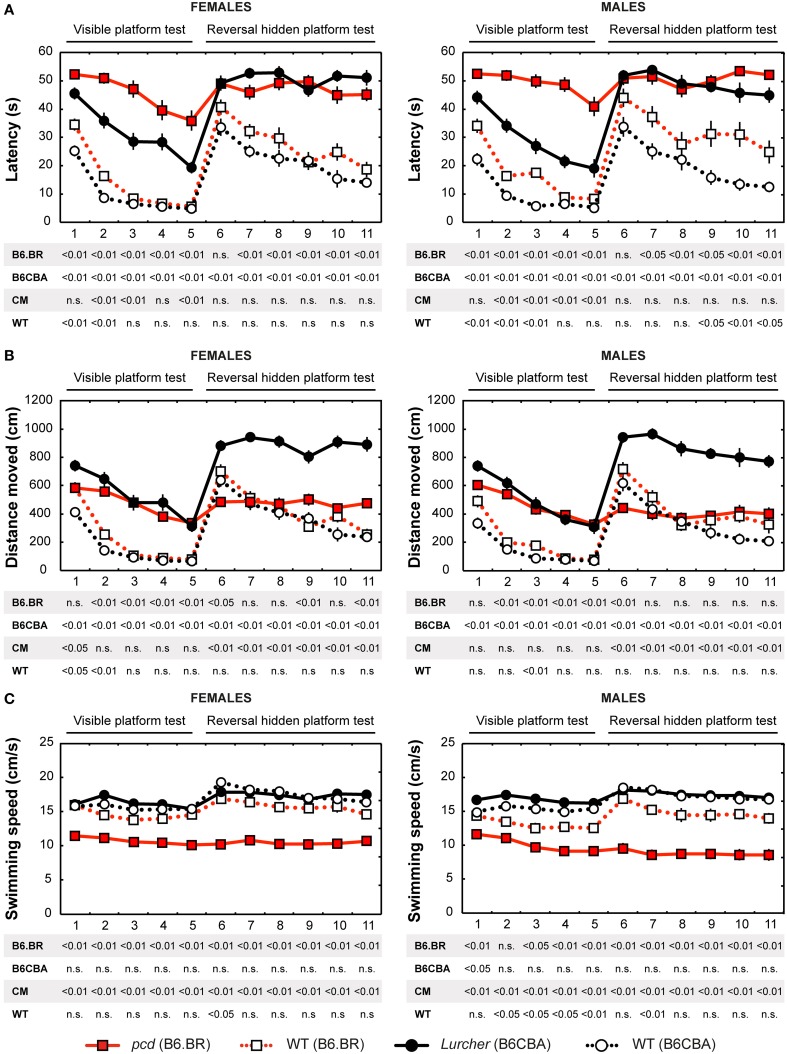
**Morris water maze: (A) Mean escape latency (s); (B) Total distance moved (cm) and (C) Mean swimming speed during periods of active swimming (cm/s)**. Statistical significance was evaluated using permutational *t*-test with Bonferroni correction for the repeated measurement for within-strain comparisons (*pcd* vs. B6.BR wild type, *Lurcher* vs. B6CBA wild type) as well as for between-strain comparisons of cerebellar mutants (CM; *pcd* vs. *Lurcher*) and wild types (WT; B6.BR WT vs. B6CBA WT). Data are presented as mean ± SEM.

**Figure 4:**
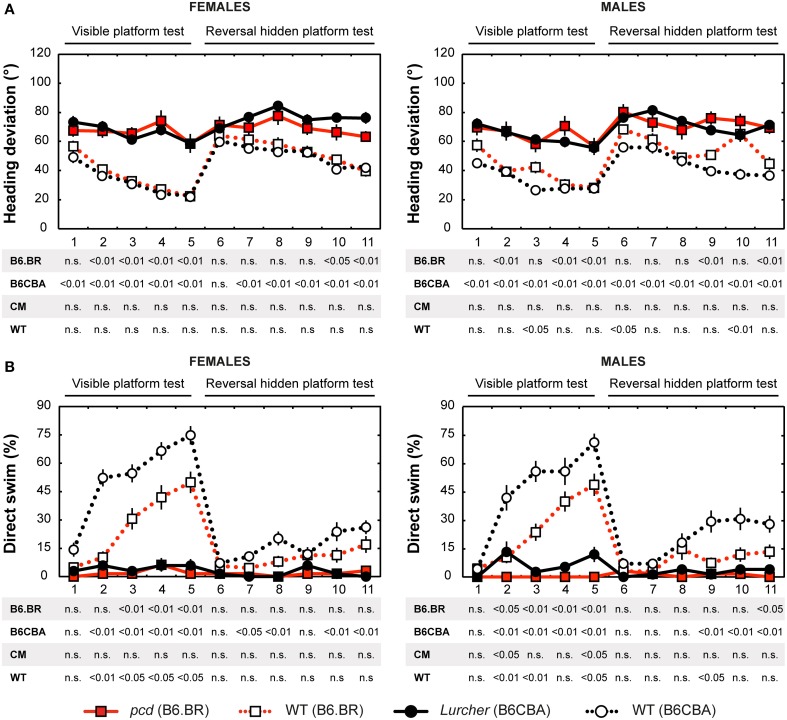
**Morris water maze: (A) Heading deviation (°) from the direct swim to the platform and (B) Percentage of direct swim trials (%)**. Statistical significance was evaluated using permutational *t*-test with Bonferroni correction for the repeated measurement for within-strain comparisons (*pcd* vs. B6.BR wild type, *Lurcher* vs. B6CBA wild type) as well as for between-strain comparisons of cerebellar mutants (CM; *pcd* vs. *Lurcher*) and wild types (WT; B6.BR WT vs. B6CBA WT). Data are presented as mean ± SEM.

**Figure 5:**
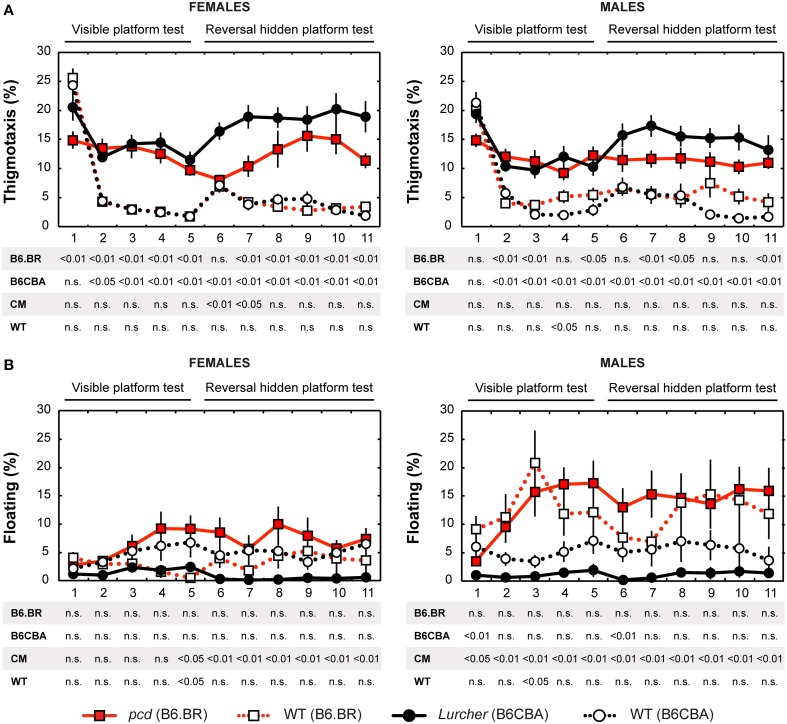
**Morris water maze: (A) Percentage of thigmotaxis (% of distance moved in 10 cm border zone) and (B) Percentage of floating (% of time spent without swimming)**. Statistical significance was evaluated using permutational *t*-test with Bonferroni correction for the repeated measurement for within-strain comparisons (*pcd* vs. B6.BR wild type, *Lurcher* vs. B6CBA wild type) as well as for between-strain comparisons of cerebellar mutants (CM; *pcd* vs. *Lurcher*) and wild types (WT, B6.BR WT vs. B6CBA WT). Data are presented as mean ± SEM.

**Figure 6:**
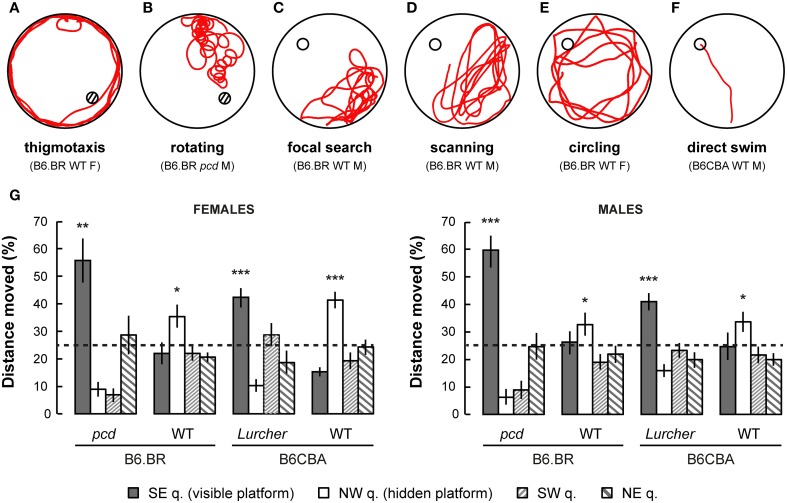
**Examples of typical trajectories (A–F) of cerebellar mutants and wild type mice. (A)** Thigmotaxis (e.g., B6.BR WT female, day-session 1, trial 1) was typical for *Lurcher* (Lc) and both B6.BR and B6CBA wild type (WT) mice on the first day-session. **(B)** Rotation (e.g., *pcd* male, day-session 1, trial 1) was an abnormal trajectory pattern that occurred in both cerebellar mutants in all day-sessions. **(C)** Focal search (e.g., B6.BR WT male, day-session 6, trial 1) in area of previous platform position appeared in WT and *Lc* mice immediately after change of the platform position (day-session 6). **(D)** Scanning (e.g., B6.BR WT male, day-session 6, trial 3) or **(E)** circling (e.g., B6.BR WT female, day-session 7, trial 1) strategy was used by WT mice before they learned the new platform position. **(F)** Direct swim (e.g., B6CBA WT male, day-session 11, trial 3) typical for WT mice in day-sessions 2–5 (visible platform) and appearing also in WT mice at the end of hidden platform task, but not in cerebellar mutants. Hatched circles indicate position of visible platform empty circles indicate position of hidden platform. **(G)** Morris water maze (probe trial): Percentage of distance moved through individual quadrants during the first 30 s of the first trial (E-starting position) of day-session 12. Statistical significance was evaluated using one-sample permutational *t*-test against 25% value, which represents a random occurrence: ^*^p < 0.05 and ^**^p < 0.01, ^***^p < 0.001. Data are presented as mean ± SEM.

Parameters measured in the Morris water maze are displayed in Figures 3–5. For the significance of the effect of individual factors (type, strain, sex, day-session) and their interactions on parameters measured in the Morris water maze, see Tables 3, 4. Typical examples of trajectory shapes observed during the experiment are shown in **Figures 6A–F**.

**Table 3:** **Morris water maze—escape latency, distance moved, and swimming speed: statistical significances of the between-group factors (type, strain, and sex) and within-group factors (session) as well as their interactions**.

**VISIBLE PLATFORM TASK**						
**Between-group factors**	**Latency**		**Distance moved**		**Swimming speed**	
	***F*_(1, 138)_**	***p***	***F*_(1, 138)_**	***p***	***F*_(1, 138)_**	***p***
Type	665.03	<0.001	424.95	<0.001	15.29	<0.001
Strain	100.00	<0.001	0.01	n.s.	148.94	<0.001
Sex	0.70	n.s.	1.18	n.s.	2.41	n.s.
Type:Strain	31.43	<0.001	15.00	<0.001	58.87	<0.001
Type:Sex	0.11	n.s.	0.03	n.s.	1.62	n.s.
Strain:Sex	4.91	n.s.	0.07	n.s.	3.88	0.043
Type:Strain:Sex	0.57	n.s.	0.17	n.s.	0.01	n.s.
**Within-group factors**	***F*_(4, 552)_**	***p***	***F*_(4, 552)_**	***p***	***F*_(4, 552)_**	***p***
Session	221.38	<0.001	251.08	<0.001	13.52	<0.001
Type:Session	15.50	<0.001	17.14	<0.001	2.22	n.s.
Strain:Session	1.66	n.s.	0.47	n.s.	7.08	<0.001
Sex:Session	1.62	n.s.	0.95	n.s.	0.90	n.s.
Type:Strain:Session	14.23	<0.001	11.25	<0.001	0.95	n.s.
Type:Sex:Session	0.68	n.s.	2.79	<0.001	0.09	n.s.
Strain:Sex:Session	2.01	n.s.	0.80	n.s.	0.21	n.s.
Type:Strain:Sex:Session	2.62	0.037	1.50	n.s.	0.85	n.s.
**REVERSAL HIDDEN PLATFORM TASK**						
**Between-group factors**	**Latency**		**Distance moved**		**Swimming speed**	
	***F*_(1, 138)_**	***p***	***F*_(1, 138)_**	***p***	***F*_(1, 138)_**	***p***
Type	291.22	<0.001	192.35	<0.001	57.66	<0.001
Strain	6.71	0.005	89.06	<0.001	163.72	<0.001
Sex	0.66	n.s.	3.50	n.s.	2.72	n.s.
Type:Strain	11.03	0.008	176.79	<0.001	60.70	<0.001
Type:Sex	0.07	n.s.	0.48	n.s.	0.14	n.s.
Strain:Sex	4.45	0.025	0.01	n.s.	2.48	0.048
Type:Strain:Sex	0.05	n.s.	1.50	n.s.	0.43	n.s.
**Within-group factors**	***F*_(5, 690)_**	***p***	***F*_(5, 690)_**	***p***	***F*_(5, 690)_**	***p***
Session	28.67	<0.001	43.91	<0.001	16.82	<0.001
Type:Session	15.68	<0.001	20.09	<0.001	5.42	<0.001
Strain:Session	1.13	n.s.	1.33	n.s.	0.47	n.s.
Sex:Session	1.09	n.s.	1.11	n.s.	0.62	n.s.
Type:Strain:Session	1.21	n.s.	1.75	0.025	0.45	n.s.
Type:Sex:Session	0.13	n.s.	0.82	n.s.	0.92	n.s.
Strain:Sex:Session	2.07	n.s.	0.90	n.s.	1.05	n.s.
Type:Strain:Sex:Session	1.79	n.s.	1.26	n.s.	0.18	n.s.

*Permutational Three-Way ANOVA with repeated measurements*.

**Table 4:** **Morris water maze—heading deviation, direct swim percentage, thigmotaxis, and floating: statistical significances of the between-group factors (type, strain, and sex) and within-group factors (session) as well as their interactions**.

**VISIBLE PLATFORM TASK**								
**Between-group factors**	**Heading**		**Direct swim**		**Thigmotaxis**		**Floating**	
	***F*_(1, 138)_**	***p***	***F*_(1, 138)_**	***p***	***F*_(1, 138)_**	***p***	***F*_(1, 138)_**	***p***
Type	314.21	<0.001	553.17	<0.001	101.37	<0.001	0.29	n.s.
Strain	1.45	n.s.	80.97	<0.001	0.39	n.s.	16.35	<0.001
Sex	0.02	n.s.	2.87	n.s.	1.89	n.s.	12.13	<0.001
Type:Strain	1.17	n.s.	37.56	<0.001	1.80	n.s.	5.82	0.037
Type:Sex	2.08	n.s.	2.46	n.s.	1.78	n.s.	1.11	n.s.
Strain:Sex	0.52	n.s.	0.03	n.s.	0.73	n.s.	13.43	<0.001
Type:Strain:Sex	0.10	n.s.	2.21	n.s.	0.05	n.s.	0.42	n.s.
**Within-group factors**	***F*_(4, 552)_**	***p***	***F*_(1, 138)_**	***p***	***F*_(4, 552)_**	***p***	***F*_(4, 552)_**	***p***
Session	43.71	<0.001	75.07	<0.001	214.95	<0.001	7.79	<0.001
Type:Session	11.01	<0.001	52.12	<0.001	60.72	<0.001	3.11	0.003
Strain:Session	0.96	n.s.	7.20	<0.001	1.65	n.s.	3.61	<0.001
Sex:Session	0.23	n.s.	0.46	n.s.	3.58	n.s.	1.44	n.s.
Type:Strain:Session	2.57	0.034	3.13	<0.001	3.61	<0.001	6.90	<0.001
Type:Sex:Session	0.91	n.s.	0.19	n.s.	3.39	<0.001	0.45	n.s.
Strain:Sex:Session	0.14	n.s.	0.61	n.s.	1.20	n.s.	6.03	<0.001
Type:Strain:Sex:Session	1.44	n.s.	0.81	n.s.	0.73	n.s.	0.55	n.s.
**REVERSAL HIDDEN PLATFORM TASK**								
**Between-group factors**	**Heading**		**Direct swim**		**Thigmotaxis**		**Floating**	
	***F*_(1, 138)_**	***p***	***F*_(1, 138)_**	***p***	***F*_(1, 138)_**	***p***	***F*_(1, 138)_**	***p***
Type	174.80	<0.001	113.05	<0.001	124.96	<0.001	0.07	n.s.
Strain	0.90	n.s.	16.52	<0.001	6.76	0.008	13.07	<0.001
Sex	0.36	n.s.	0.51	n.s.	0.62	n.s.	5.87	0.018
Type:Strain	8.64	<0.001	13.26	<0.001	10.45	<0.001	6.64	<0.001
Type:Sex	0.30	n.s.	0.16	n.s.	2.31	n.s.	0.00	n.s.
Strain:Sex	6.36	0.014	1.40	n.s.	1.39	n.s.	4.40	n.s.
Type:Strain:Sex	0.02	n.s.	0.39	n.s.	0.00	n.s.	0.01	n.s.
**Within-group factors**	***F*_(5,690)_**	***p***	***F*_(5,690)_**	***p***	***F*_(5,690)_**	***p***	***F*_(5,690)_**	***p***
Session	16.00	<0.001	12.51	<0.001	3.28	n.s.	2.14	0.022
Type:Session	5.30	<0.001	9.32	<0.001	7.53	<0.001	0.51	n.s.
Strain:Session	2.77	<0.001	1.96	<0.001	1.77	n.s.	1.28	n.s.
Sex:Session	3.62	0.005	0.72	n.s.	2.07	<0.001	1.36	n.s.
Type:Strain:Session	0.82	n.s.	0.83	n.s.	0.94	n.s.	1.27	n.s.
Type:Sex:Session	1.65	n.s.	1.26	n.s.	2.65	0.024	1.23	n.s.
Strain:Sex:Session	2.78	0.013	1.26	n.s.	0.43	n.s.	1.17	n.s.
Type:Strain:Sex:Session	0.15	n.s.	2.55	0.005	3.01	0.004	0.62	n.s.

*Permutational Three-Way ANOVA with repeated measurements*.

#### Escape latencies

Escape latencies in the Morris water maze (Figure 3A) were significantly longer in both types of cerebellar mutants than in their wild type littermates during the test with the visible as well as the hidden platform. The only day on which the difference was low (for the B6.BR mice, it was insignificant) was the first day with the hidden platform moved into the opposite quadrant (D6). Strain comparison showed smaller differences in both mutant as well as wild type mice. Compared with *Lurchers, pcd* mice (both males and females) had longer latencies in the visible platform task, while no differences between the mutants were found during the hidden platform task. In addition, B6.BR wild type mice achieved worse results than their B6CBA counterparts did (for females, only at the beginning of the visible platform task, but for males also at the end of the hidden platform task). The direct comparison of females and males showed differences in B6.BR wild type mice only on the day-session 3 (*t* = −5.28, *p* < 0.001) and day-session 5 (*t* = −2.72, *p* = 0.023).

#### Distance moved

Distance moved (Figure 3B) was significantly longer in *Lurcher* mice compared with wild type B6CBA mice on all days of the test. On the other hand, *pcd* mice showed a markedly longer trajectory than wild type B6.BR mice only in the visible platform task, except for on the first day, while the difference appeared only occasionally in the next phase. In wild type mice, mild strain differences appeared only in the visible platform task. Nevertheless, in the mutants, a significant difference appeared in the hidden platform task when the distance moved was markedly longer in *Lurchers* than in *pcd* mice. Sex differences were found only on the day-session 3 in B6.BR wild type mice (*t* = −2.72, *p* = 0.037).

#### Swimming speed

Swimming speed (Figure 3C) was significantly lower in *pcd* mice than in other mice.

On the other hand, *Lurchers* did not swim slower than their wild type littermates. *Lurcher* males were even significantly faster than wild type ones on day-session 1. Strain comparison showed a slower swimming speed in B6.BR wild type mice than in B6CBA ones, namely in the visible platform phase. There were no significant sex differences in swimming speed.

#### Heading deviation

Heading deviation error (Figure 4A) was significantly higher in mutant mice than in their wild type littermates. In the B6.BR strain, the differences were mainly seen in the visible platform task, while, in the B6CBA strain, they were significant for almost the entire course of the experiment. In females, no strain differences were seen. B6.BR wild type males were occasionally worse than B6CBA males. The only sex difference in heading deviation error was found on the day-session 10 in B6.BR wild type mice (*t* = −3.42, *p* = 0.011).

#### Direct swim

The percentage of direct swim trials (Figure 4B) was high in wild type mice of both strains in the visible platform task except for the first day session. Also, on some days of the hidden platform task, wild type mice showed a significantly higher percentage of direct swim trials than their mutant littermates. In mutant mice, direct swim trials were rare in both phases of the test. Strain differences showing better performance in B6CBA mice were only seen for a few day-sessions and mainly for wild type mice. Males and females did not differ in direct swim percentage.

#### Thigmotaxis

Thigmotaxis (Figure 5A) was significantly higher in both cerebellar mutants than in wild type animals on most days of the water maze test. Strain differences, on the other hand, were poor. The only difference between males and females was found on the day-session 5 in B6.BR wild type mice (*t* = −3.19, *p* = 0.010).

#### Floating analysis

Floating analysis (Figure 5B) revealed almost no differences in the percentage of time spent without activity between mutant and wild type mice. Nevertheless, in cerebellar mutants, a strain difference was observed, since floating behavior was very rare in *Lurcher* mutants. B6.BR wild type males spent significantly more time floating than females on the day-session 3 (*t* = −3.47, *p* = 0.003), day-session 4 (*t* = −2.86, *p* = 0.015), day-session 5 (*t* = −2.87, *p* = 0.013), and day-session 7 (*t* = −2.87, *p* = 0.035).

#### Morris water maze task acquisition

Significance of the within factor (day-session) indicated the importance of development of the parameters during the course of the Morris water maze test (Tables 3, 4). Learning process, which was manifested as a shortening of escape latencies (Figure 3A, Table 5) and distance moved (Figure 3B, Table 5), was detectable for the visible platform task in all groups of mice and for the hidden platform task for both B6CBA and B6.BR wild type mice and B6CBA *Lurcher* males. *Lurcher* females and both *pcd* males and females did not learn the hidden platform task (Table 5). Wild type mice of both strains also showed a significant decrease of heading deviation error (Figure 4A, Table 5) and an increase of the direct swim percentage (Figure 4B, Table 5) during both visible and hidden platform tasks. *Lurcher* mice improved heading deviation and direct swim percentage (males only) during the visible platform task, but not during the hidden platform task. *Pcd* mice did not improve their heading deviation or direct swim percentage in either of the tasks.

**Table 5:** **Morris water maze: statistical significances of change between or during individual phases**.

**VISIBLE PLATFORM TASK (DAY-SESSION 1 VS. DAY-SESSION 5)**								
	**Latency**		**Distance moved**		**Heading**		**Direct swim**	
**Females**	***t***	***p***	***t***	***p***	***t***	***p***	***t***	***p***
*pcd* B6.BR	4.90	<0.001	7.43	<0.001	1.39	n.s.	−1	n.s.
Wild type B6.BR	14.25	<0.001	13.28	<0.001	9.46	<0.001	−7.51	<0.001
*Lurcher* B6CBA	8.82	<0.001	7.83	<0.001	3.06	0.007	−0.81	n.s.
Wild type B6CBA	11.44	<0.001	9.52	<0.001	4.93	<0.001	−9.55	<0.001
**Males**	***t***	***p***	***T***	***p***	***t***	***p***	***t***	***p***
*pcd* B6.BR	3.80	0.002	7.99	<0.001	2.06	n.s.	0	n.s.
Wild type B6.BR	9.54	<0.001	9.26	<0.001	5.50	<0.001	−6.67	<0.001
*Lurcher* B6CBA	9.23	<0.001	8.74	<0.001	3.68	0.001	−2.96	0.016
Wild type B6CBA	7.27	<0.001	6.68	<0.001	5.11	<0.001	−14.75	<0.001
**REVERSAL HIDDEN PLATFORM TASK (DAY-SESSION 6 VS. DAY-SESSION 11)**								
	**Latency**		**Distance moved**		**Heading**		**Direct swim**	
**Females**	***t***	***p***	***T***	***p***	***t***	***p***	***t***	***p***
*pcd* B6.BR	1.14	n.s.	0.22	n.s.	1.17	n.s.	−0.56	n.s.
Wild type B6.BR	6.00	<0.001	7.52	<0.001	4.09	<0.001	4.55	<0.001
*Lurcher* B6CBA	−0.58	n.s.	−0.15	n.s.	−1.47	n.s.	1.73	n.s.
Wild type B6CBA	6.23	<0.001	7.27	<0.001	4.35	<0.001	11.12	<0.001
**Males**	***t***	***p***	***t***	***p***	***t***	***p***	***t***	***p***
*pcd* B6.BR	−0.43	n.s.	1.12	n.s.	1.96	n.s.	0	n.s.
Wild type B6.BR	3.63	0.004	4.38	<0.001	3.66	0.002	7.32	<0.001
*Lurcher* B6CBA	3.16	0.004	3.65	0.002	1.41	n.s.	1.68	n.s.
Wild type B6CBA	5.62	<0.001	6.22	<0.001	4.50	<0.001	7.62	<0.001
**PLATFORM TRANSITION (DAY-SESSION 5 VS. DAY-SESSION 6)**								
	**Latency**		**Distance moved**		**Heading**		**Direct swim**	
**Females**	***t***	***p***	***t***	***p***	***t***	***p***	***t***	***p***
*pcd* B6.BR	−3.23	0.006	−2.93	0.011	−1.78	n.s.	0	n.s.
Wild type B6.BR	−13.71	<0.001	−13.16	<0.001	−15.43	<0.001	7.8	<0.001
*Lurcher* B6CBA	−8.76	<0.001	−9.28	<0.001	−2.51	0.025	1.14	n.s.
Wild type B6CBA	−10.42	<0.001	−12.56	<0.001	−8.48	<0.001	11.78	<0.001
**Males**	***t***	***p***	***t***	***p***	***t***	***p***	***t***	***p***
*pcd* B6.BR	−2.64	0.020	−2.19	0.042	−2.80	0.016	−1.46	n.s.
Wild type B6.BR	−11.81	<0.001	−12.63	<0.001	−10.13	<0.001	7.91	<0.001
*Lurcher* B6CBA	−8.55	<0.001	−8.35	<0.001	−4.55	<0.001	2.96	0.016
Wild type B6CBA	−8.36	<0.001	−9.60	<0.001	−6.91	<0.001	13.83	<0.001

*Permutational paired t-test*.

Change of the platform position and its concealment (compare day-sessions 5 and 6) led to significant prolongation of both latencies and distance moved in all groups of mice (Figures 3A,B, Table 5), an increase in heading deviation error in all groups except *pcd* females (Figure 4A, Table 5) and a decrease in the direct swim percentage in wild type mice and *Lurcher* males (Figure 4B, Table 5). The effect of change of the platform position and its concealment is also shown in Supplementary Figure 1.

#### Probe trial

Probe trial on the last day of the Morris water maze test showed a mild preference for the NW quadrant in which the hidden platform was localized for the previous 6 day-sessions in B6CBA and B6.BR wild type mice. Surprisingly, both types of cerebellar mutants showed a significant preference for the SE quadrant, where the visible platform was localized during the first phase of the water maze test (Figure 6G). These findings were confirmed by the measurement of latency of the first occurrence in the former position of the visible and hidden platform (Supplementary Figure 2).

### Forced swimming test

**Figure 7:**
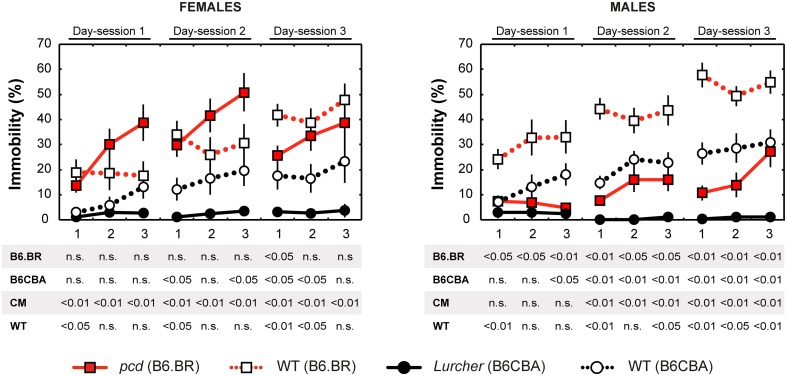
**Forced swimming test: percentage of immobility state (% of time spent without moving)**. Statistical significance was evaluated using a permutational *t*-test with a Bonferroni correction for the repeated measurement for within-strain comparisons (*pcd* vs.B6.BR wild type, *Lurcher* vs. B6CBA wild type) and for between-strain comparisons of cerebellar mutants (CM; *pcd* vs. *Lurcher*) and wild types (WT; B6.BR WT vs. B6CBA WT). Sex differences were significant in *pcd* mutants only from day-session 1, time-bout 2 until day-session 3, time-bout 1 (*t* = 3.13, *p* = 0.011; *t* = 4.05, *p* = 0.003; *t* = 3.89, *p* = 0.004; *t* = 2.90, *p* = 0.030; *t* = 3.67; *p* = 0.005; *t* = 2.93, *p* = 0.019, respectively). Data are presented as mean ± SEM.

Depressive-like behavior, which manifested as a state of immobility in the Porsolt's forced swimming test, is presented in Figure 7. The analysis showed a significant between-group effect of the type and strain, but not their interaction (Table 6). Nevertheless, a Three-Way ANOVA showed a significant effect of type:sex as well as type:strain:sex factor interactions (Table 6). A repeated measurement ANOVA also showed a significant within-group factor effect of the time-bout and day-session (Table 6).

**Table 6:** **Forced swimming test: statistical significances of the between-group factors (type, strain, and sex) and within-group factors (bout, session) as well as their interactions**.

	**Immobility**	
**Between-group factors**	***F*_(1, 99)_**	***p***
Type	39.45	<0.001
Strain	76.79	<0.001
Sex	0.03	n.s.
Type:Strain	0.43	n.s.
Type:Sex	18.15	<0.001
Strain:Sex	2.10	n.s.
Type:Strain:Sex	7.17	0.013
**Within-group factors**	***F*_(2, 198)_**	***p***
Bout	23.02	0.011
Type:Bout	2.65	0.025
Strain:Bout	1.69	n.s.
Sex:Bout	1.37	n.s.
Type:Strain:Bout	17.60	<0.001
Type:Sex:Bout	2.87	0.023
Strain:Sex:Bout	1.13	n.s.
Type:Strain:Sex:Bout	1.67	n.s.
**Within-group factors**	***F*_(1, 99)_**	***p***
Session	29.48	<0.001
Type:Session	12.91	<0.001
Strain:Session	5.00	0.005
Sex:Session	0.72	n.s.
Type:Strain:Session	0.24	n.s.
Type:Sex:Session	0.27	n.s.
Strain:Sex:Session	0.10	n.s.
Type:Strain:Sex:Session	0.03	n.s.
**Within-group factors**	***F*_(2, 198)_**	***p***
Bout:Session	1.83	n.s.
Type:Bout:Session	1.08	n.s.
Strain:Bout:Sesion	1.22	n.s.
Type:Strain:Bout:Session	1.15	n.s.
Sex:Bout:Session	0.30	n.s.
Type:Sex:Bout:Sesion	3.56	n.s.
Strain:Sex:Bout:Sesion	0.41	n.s.
Type:Strain:Sex:Bout:Session	2.63	n.s.

*Permutational Three-Way ANOVA with repeated measurements*.

Total length of immobility in *pcd* females did not significantly differ from that in B6.BR wild type females (except the first 5 min time-bout in day-session 3). On the contrary, *pcd* males showed less immobility than B6.BR wild type males (Figure 7). In B6CBA mice, both *Lurcher* females and males had a shorter duration of immobility than did wild type mice in most time-bouts of day-session 2 and 3 (Figure 7). The occurrence of immobility periods was very low in *Lurcher* mice; thus, their immobility state duration was significantly shorter than in *pcd* mice in each day-session for females and the last 2 day-sessions for males. B6CBA wild type females showed less immobility than B6.BR wild type females in the first time-bout for day-session 1 and 2 as well as the first two time-bouts on day-session 3. The sex differences were found in *pcd* mice; males showed less immobility than females (Figure 7).

Furthermore, while the immobility was permanently rare in *Lurchers*, its duration increased from the day-session 1 to 3 in other mice (Table 7). For pair comparison of time-bout 1 vs. time-bout 3 for each day-session, see Supplementary Table 2.

**Table 7:** **Forced swimming test: paired comparison of day-session 1 and 3 for each time-bout**.

**Groups**	**Time-bout (min)**	**Females**		**Males**	
		***t***	***p***	***t***	***p***
*pcd* B6.BR	00-05	−4.60	<0.001	−0.86	n.s.
	05-10	−0.53	n.s.	−1.44	n.s.
	10-15	0.01	n.s.	−3.22	0.006
Wild type B6.BR	00-05	−4.07	0.003	−7.48	<0.001
	05-10	−3.30	0.012	−2.46	0.028
	10-15	−5.17	0.001	−2.93	0.011
*Lurcher* B6CBA	00-05	−1.22	n.s.	1.04	n.s.
	05-10	0.14	n.s.	0.66	n.s.
	10-15	−0.51	n.s.	0.60	n.s.
Wild type B6CBA	00-05	−2.93	0.011	−4.24	<0.001
	05-10	0.14	n.s.	0.66	n.s.
	10-15	−1.36	n.s.	−2.41	0.033

*Permutational paired t-test*.

### Quantitative histology

**Figure 8:**
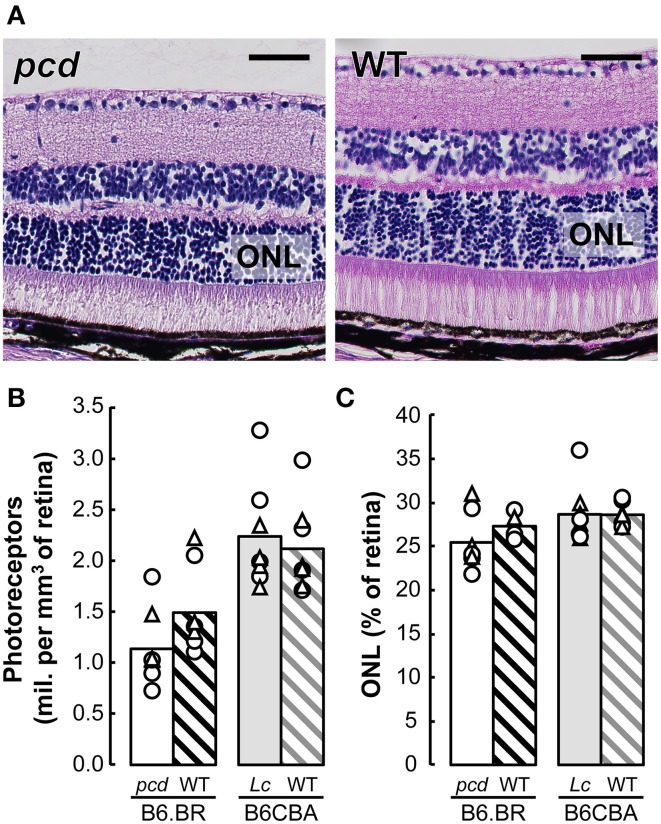
**(A)** Histology of the retina in *pcd* and B6.BR wild type (WT) mice stained with Gill's hematoxylin. The bars indicate 50 μm. **(B)** Mean number of photoreceptors in outer nuclear layer (ONL) per mm^3^ of the retina and **(C)** mean volume of ONL expressed as percentage of whole retina volume in *pcd*, B6.BR WT, *Lurcher* (Lc) and B6CBA WT mice. Values of individual animals are indicated by Δ for females and ◯ for males.

Stereological analysis showed only an insignificant reduction in the density of retinal photoreceptors nor ONL volume relative to whole retina volume in *pcd* mutants compared with their healthy littermates, or with B6CBA *Lurcher* and wild type mice (Figure 8).

## Discussion

In this study, we have shown specific features of spatial performance and behavioral differences in response to the Morris water maze task in *pcd* and *Lurcher* mice, the most frequently used mouse models of olivocerebellar degeneration (for review, see Lalonde and Strazielle, 2007; Cendelin, 2014). Although it has been described that the neurodegenerative process disrupts spatial learning in both mutants, specific features of their spatial performance, which are presented here, have not been reported and sufficiently explained before, and even contradict some earlier opinions (Goodlett et al., 1992; Lalonde and Thifault, 1994). With regard to many factors that could influence the performance of the mice in behavioral tests, e.g., specific mutations, genetic background, sex, and the environment (for review, see Wolfer and Lipp, 2000; D'hooge and de Deyn, 2001), we performed a detailed comparative behavioral analysis of spatial navigation, learning and memory in *pcd* and *Lurcher* mutants. In order to assess specific behavioral abnormalities that could influence performance in the spatial navigation task, open field and forced swimming tests were done.

### Behavior of *Pcd* and *Lurcher* mutants in the open field and forced swimming test

The type of the mutation, background strain and sex influenced behavior in both open field and forced swimming tests. The effect of sex of experimental animals was relatively stronger in these tests than in the Morris water maze and was, in this case, sufficient to completely invert the differences. In the open field, B6.BR mice showed typical preference to the corners of the arena, while, in B6CBA mice, the activity was more dispersed through the arena. Such higher dispersion of the activity was more marked in *Lurchers* than in their wild type littermates. Abnormal exploration has been reported also by Caston et al. (1998) who found significantly reduced exploratory behavior in *Lurchers* despite an increase in spontaneous activity. The most obvious phenomenon observed in the forced swimming test was the absence of floating even during later phases of each day-session of the forced swimming test and the absence of an increase of floating duration across the day-sessions in *Lurcher* mice. While tendency toward inactivity and depressive-like behavior were observed in *pcd* mice, *Lurchers* showed rather inadequate high activity.

Features of behavior seen in both open field and forced swimming tests comply with behavioral disinhibition affecting *Lurcher* mice (Frederic et al., 1997; Lalonde, 1998; Hilber et al., 2004; Porras-Garcia et al., 2005). The discrepancy between less fear-related behavior and elevated levels of corticosterone during stressful situations (Frederic et al., 1997; Hilber et al., 2004; Lorivel et al., 2014), a lack of prepulse inhibition and an inability to produce the immobility response suggest that *Lurcher* mice have a reduced capacity to inhibit selective components of natural behaviors due to an affection of the sensorimotor gating mechanism (Lalonde, 1998; Porras-Garcia et al., 2005). In *pcd* mice, only indirect evidence suggesting the possibility of some level of behavioral disinhibition and perseveration were reported in studies of spontaneous alternation, exploration and habituation (Lalonde et al., 1987, 1989). Since more dispersed activity in the open field and less frequent immobility in the forced swimming test were also in B6CBA wild type mice as compared with B6.BR wild type mice, these phenomena are not only due to the *Lurcher* phenotype, but might be at least a partially strain-related phenomenon.

### Performance of *Pcd* and *Lurcher* mutants in the morris water maze tests

Both *pcd* and *Lurcher* cerebellar mutants showed poor performance in the Morris water maze. Despite finding a marked improvement in *Lurcher* mice during the visible platform task, the results were worse than in wild type controls, and there were only a few trials with a direct swim toward the goal. The results for learning the hidden platform position were much worse, and among cerebellar mutants, it was detectable only in *Lurcher* males. This complies with earlier findings (Lalonde and Thifault, 1994; Cendelin et al., 2014), and may support the hypothesis that *Lurcher* mice have impaired visuomotor integration suggested by Lalonde and Thifault (1994). Nevertheless, visuomotor integration ability seems to be partially preserved in *Lurchers*, since they are able to learn the visual platform task. Preference for the zone of the previous platform position during the first trial after changing the platform position (analogy of probe trial) also supports the idea that *Lurcher* mutants have some level of spatial learning ability that seems to be strongly dependent on the possibility of visual guidance training, which was constituted in our study by the 5 day-sessions of the visual platform task. Poor performance in the hidden platform task, on the other hand, may suggest a severe spatial learning deficit.

Although it has been reported in *pcd* mice that their performance in the visual platform task is not impaired compared to wild type controls (Goodlett et al., 1992), we observed poor performance of *pcd* mice in both visual and hidden platform tasks. The first study of spatial navigation in *pcd* mice used quite small experimental groups of male mice (Goodlett et al., 1992). In the present study, the results are based on larger samples, and males and females were analyzed separately.

An interesting phenomenon seen in the probe trial was the marked preference for the quadrant in which the visible platform was localized, and the omitting of the quadrant of the more recent localization of the hidden platform in both types of cerebellar mutants. In *pcd* mice, this is an artifact of spending a long time in the proximity of the starting point due to low activity. Furthermore, the reversal hidden platform task seemed to be extremely difficult for *Lurchers*. The preference of the original target quadrant could be explained by the behavioral inflexibility of *Lurcher* mice (Dickson et al., 2010). Behavioral flexibility, inhibitory response, and working memory are high-level cognitive skills, which enable the effective execution of goal-directed behaviors (Dalley et al., 2004). These skills have consistently been shown to be dependent on the prefrontal cortex (Dalley et al., 2004; Robbins and Arnsten, 2009). It has been demonstrated that the cerebellum modulates the prefrontal cortex activity (Strick et al., 2009; Rogers et al., 2013). Behavioral inflexibility, as well as behavioral disinhibition, which are closely related to inhibitory response (Young et al., 2009), suggested the affection of higher cognitive skills in *Lurcher* mutants. Thus, the poor performance of cerebellar mutants in the water maze task could be caused by at least four types of factors or their combinations: (1) Cognitive disorders, (2) Sensory disorders, (3) Motivation and behavioral abnormalities, and (4) Motor deficits. The performance is further modified by differences in manifestation of the mutations, strain, and sex.

#### Role of cognition

The Morris water maze task requires at least two types of non-motor learning. First, association between the platform and escape from the maze must be created. Second, the animal must start to learn the position of the platform. Associative learning processes are supposed to be strongly related to the cerebellum (Gruart et al., 1997; Jimenez-Diaz et al., 2004; for review, see Thompson and Steinmetz, 2009; Perciavalle et al., 2013) and its abnormalities have been described in both *Lurcher* and *pcd* mutants (Chen et al., 1996; Porras-Garcia et al., 2005, 2010; Brown et al., 2010). Thus, delayed association between the platform and water escape could affect the motivation to learn its position in cerebellar mutants.

Traditionally, spatial cognition is related to the hippocampus (O'Keefe and Nadel, 1978). Nevertheless, the cerebellum participates in the construction of hippocampal spatial representation and, thus, plays an important role in goal-directed navigation (Rochefort et al., 2011, 2013; Onuki et al., 2013). Therefore, it could be assumed that the absence of Purkinje cells in both *pcd* and *Lurcher* mice might have a strong impact on the hippocampal processes involved in solving spatial tasks.

#### Role of sensory impairments

Since good vision is crucial for spatial navigation, retinal degeneration could be an important factor that strongly influences behavior, namely spatial performance in *pcd* mice. Nevertheless, the retinal degeneration in *pcd* mice is only slowly progressive (Blanks et al., 1982; Lavail et al., 1982; Blanks and Spee, 1992; Marchena et al., 2011), and we have found only an insignificant reduction of photoreceptor density in the retinas of *pcd* mice at the age at which they were tested for spatial orientation. Despite this, some impact of vision problems on behavior during spatial tasks could not be excluded due to the possible functional imperfection of a degenerating retina even before a reduction of photoreceptor number becomes evident (Marchena et al., 2011).

However, in addition, the cerebellar disorder itself may lead to severe sensory dysfunctions by at least two mechanisms—affection of perceptual processes and oculomotor abnormalities. The cerebellum is associated with perceptual systems including vision, proprioception and self-motion perception, and cerebellar lesions lead to a wide range of sensory impairments (for review, see Baumann et al., 2015). Therefore, cerebellar disorders may severely affect spatial orientation ability due to the inappropriate acquisition and processing of information necessary for space navigation.

Control of oculomotor function is important for sighting fixation and for the visual following of an object by a moving individual. In *Lurchers*, abnormalities of the optokinetic and vestibuloocular reflexes were described by van Alphen et al. (2002). Since cerebellar Purkinje cells control oculomotor coordination, including optokinetic and vestibuloocular reflexes (for review, see Angelaki and Hess, 2005; Yakusheva et al., 2007), oculomotor problems could be expected also in *pcd* mice, in which, however, the vestibuloocular reflex has been found to be almost normal (Killian and Baker, 2002).

For all of these reasons, sensory problems can be expected to play a significant role in navigation difficulties in cerebellar mutants. In the visible goal task, these problems may be less important than in the case of the hidden goal task, since the goal represents a single and marked intramaze object of interest instead of multiple extramaze landmarks necessary for hidden goal location.

#### Role of motivation and behavioral abnormalities

Paradoxically, *pcd* mice showed short distances moved. This fact can be explained by low swimming activity and longer periods of floating compared with *Lurchers*. Therefore, their trajectory was relatively short, even in the case where they did not reach the platform and spent the entire trial slowly swimming with floating periods, while *Lurcher* mice spent this time intensively swimming. Higher tendency of inactivity in *pcd* mice and higher swimming activity in *Lurche*r mice were also seen in the forced swimming test. Floating is a behavioral phenomenon that may substantially influence the results of the water maze tasks (Llano Lopez et al., 2010) or may be a response to a difficult task as a manifestation of depressive-like behavior and learned helplessness (Porsolt et al., 1979). Potential sight impairment due to retinal degeneration and poor fitness related to low body weight may make the spatial task too difficult for *pcd* mice, which might induce learned helplessness.

#### Role of motor impairment

For performance in the water maze task, swimming and direction maintenance abilities are required. Motor impairment has been shown many times in cerebellar mutants (Fortier et al., 1987; Lalonde et al., 1996; Le Marec and Lalonde, 1997, 1998; Cendelin et al., 2008, 2014). On the other hand, Fortier et al. (1987) showed a normal EMG pattern in swimming *Lurcher* mice, but not in walking ones, suggesting that swimming is not as affected by the ataxia as gait. Furthermore, *Lurcher* mice achieved the same swimming speed as wild type mice. In *pcd* mice, low swimming speed could account for their abnormal swimming pattern (Goodlett et al., 1992), but also for lower activity or worse fitness. Nevertheless, in both *Lurcher* and *pcd* mice, we have observed a high incidence of rotating, but almost no direct swim trials. A low frequency of direct swim was even seen in *Lurcher* mice at the end of the visible platform task when they showed an improving ability to reach the visible goal. Therefore, motor deficiency does not seem to affect swimming ability, but rather, could influence trajectory shape and disable the maintenance of a straight course toward the goal in cerebellar mutants.

#### Role of the mutation, strain background and sex

Poor spatial performance is a strong phenotypic manifestation of particular mutations in *pcd* and *Lurcher* mice. These symptoms are easily detectable by the tests, and other factors, such as strain and sex, seem to only slightly modulate performance. Sex dimorphism as a function of brain structures related to both behavioral processes and motor control has been described (Arvidsson et al., 2014), and significant sex differences were even reported in neurological manifestations of mutations in mice (Walton et al., 2012; Truong et al., 2013).

More problematic is the comparison of the manifestation of mutations. Despite the main features and extent of cerebellar degeneration being similar, *pcd* and *Lurcher* mice differ in a number of aspects. The overall performance of *pcd* mice in the Morris water maze was worse than in *Lurchers*. *Grid2^Lc^* and *Agtpb1^pcd^* mutations not only differ in the mechanism of cell death activation, but the spectrum of extracerebellar brain damage and the affection of other tissues was wider with the *Agtpb1^pcd^* mutation. Therefore, in this case, modifying factors have a broader range of targets. Particularly, retinal degeneration (Blanks et al., 1982; Lavail et al., 1982; Blanks and Spee, 1992; Marchena et al., 2011) and expression of *Nna1* in the skeletal muscles (Harris et al., 2000) are important. Since strain differences between B6CBA and B6.BR wild type mice were also observed in the present study, genetic background plays a role. *Pcd* and *Lurcher* mice are not commercially available on the same strain background. Therefore, it is difficult to unambiguously distinguish a specific mutation effect from the modifying effect of strain-specific phenotypic traits, the importance of which was particularly shown for floating behavior.

Recently, we have shown that, despite the *Lurcher* mutation having a strong manifestation, the phenotype could be modulated by genetic background (Cendelin et al., 2014). Considering the magnitude of differences between the same mutants in different strains and between different mutants, we could conclude that strain differences could be sufficient to cover or mitigate some of the mutation-related differences.

## Conclusion

We have confirmed the severe impairments in cognitive and behavioral tests in both *pcd* and *Lurcher* mutant mice. Contrary to previous studies (Goodlett et al., 1992; Lalonde and Thifault, 1994), we found that visuomotor integration is only partially disabled in *Lurchers*, and that *pcd* mice failed in both visual and hidden goal tests, using large samples of mice. Overall performance in the Morris water maze test was better in *Lurcher* mutants than in *pcd* mice. The effect of the mutation as well as of the genetic background was seen. The deficit of spatial performance in cerebellar mutants may potentially arise from a combination of cognitive, sensory, emotional, and motor disturbances, all of which are expected to be of different importance in various mutants. Mutation-related differences could be potentiated by specific phenotypic traits of different strains of origin than these mutants.

### Conflict of interest statement

The authors declare that the research was conducted in the absence of any commercial or financial relationships that could be construed as a potential conflict of interest.
