# Supplementary material for: Mutation-related differences in exploratory, spatial, and depressive-like behavior in pcd and Lurcher cerebellar mutant mice
Source: Front Behav Neurosci. 2015 May 12;9:116. doi: 10.3389/fnbeh.2015.00116 (PMC4429248; doi:10.3389/fnbeh.2015.00116)
Supplement: Supplementary file 1 [file Image1.PDF]

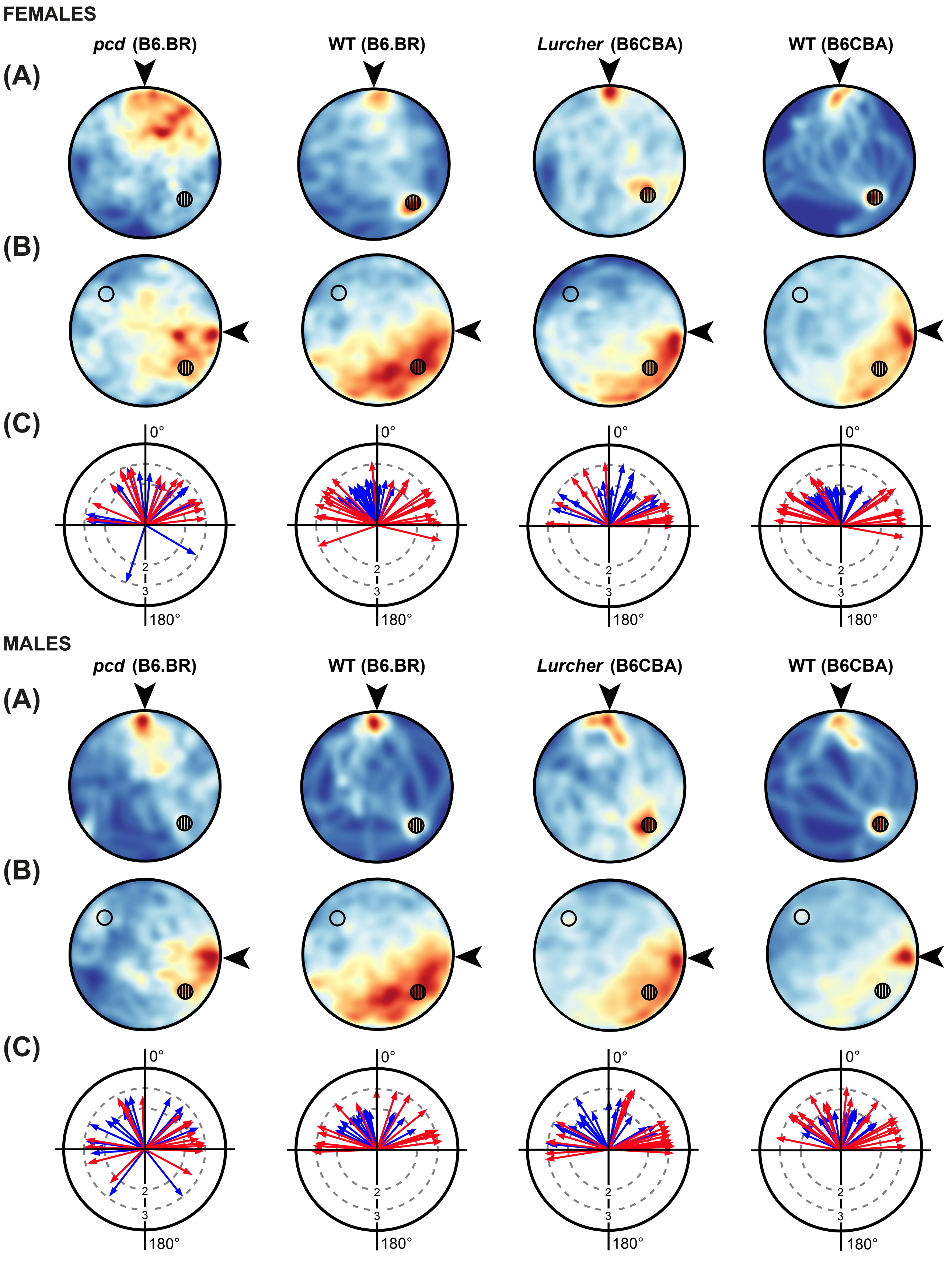
FEMALES
pcd (B6.BR)
WT (B6.BR)
Lurcher (B6CBA)
WT (B6CBA)
(A)
(B)
(C)
0°
2
3
180°
MALES
pcd (B6.BR)
WT (B6.BR)
Lurcher (B6CBA)
WT (B6CBA)
(A)
(B)
(C)
0°
2
3
180°

Supplementary Figure 1 - Effect of transition between visible platform and reversal hidden platform task in the Morris water maze. (A) Superposition of trajectories in the first trial (N-starting position depicted with arrows) of the day-session 5 (visible platform task). Hatched circles indicate the position of the visible platform. B6.BR wild type mice and both B6CBA *Lurcher* and wild type mice showed either direct or indirect swim toward the visible platform swim to the visible platform, while *pcd* mice spent most of the time floating in the start area. (B) Superposition of trajectories in the first trial (E-starting position depicted with arrows) of day-session 6 (reversal hidden platform task). Empty circles indicate current position of the hidden platform and hatched circles indicate the previous position of visible platform (visible platform task). Mice of each experimental group showed a preference for the SE quadrant with previous localization of the visible platform. Nevertheless, in *pcd* mice the area of maximum activity was focused rather in the proximity of the starting point, while in wild type mice and in *Lurchers* the center of their activity corresponded more with the previous platform position. (C) Rose graphs show trajectory vector dispersion. Blue vectors demonstrate trajectories in the first trial of day-session 5 and red vectors demonstrate trajectories in the first trial of day-session 6. Lengths of the vectors indicate distance moved in logarithmic scale (dashed circles). Directions of the vectors indicate mean deviation from direct course toward the platform (0° represents direct swim). Higher dispersion of the heading direction vectors and longer trajectories in *pcd* mice at the end of the visible platform task (day-session 5), as well as immediately after changing the platform position and its concealment (day-session 6) suggest that *pcd* mice did not search the platform in the appropriate area despite spending a lot of time there (A, B). In *Lurcher* mice, both vector direction dispersion and length in the visible platform task (day-session 5) were lower than in *pcd* mice but higher than in B6CBA and B6.BR wild type mice. The difference in vector direction dispersion and length between day-sessions 5 and 6 was also much more marked in *Lurcher* and both wild type mice than in *pcd* mice.
