# Supplementary material for: Mutation-related differences in exploratory, spatial, and depressive-like behavior in pcd and Lurcher cerebellar mutant mice
Source: Front Behav Neurosci. 2015 May 12;9:116. doi: 10.3389/fnbeh.2015.00116 (PMC4429248; doi:10.3389/fnbeh.2015.00116)
Supplement: Supplementary file 2 [file Image2.PDF]

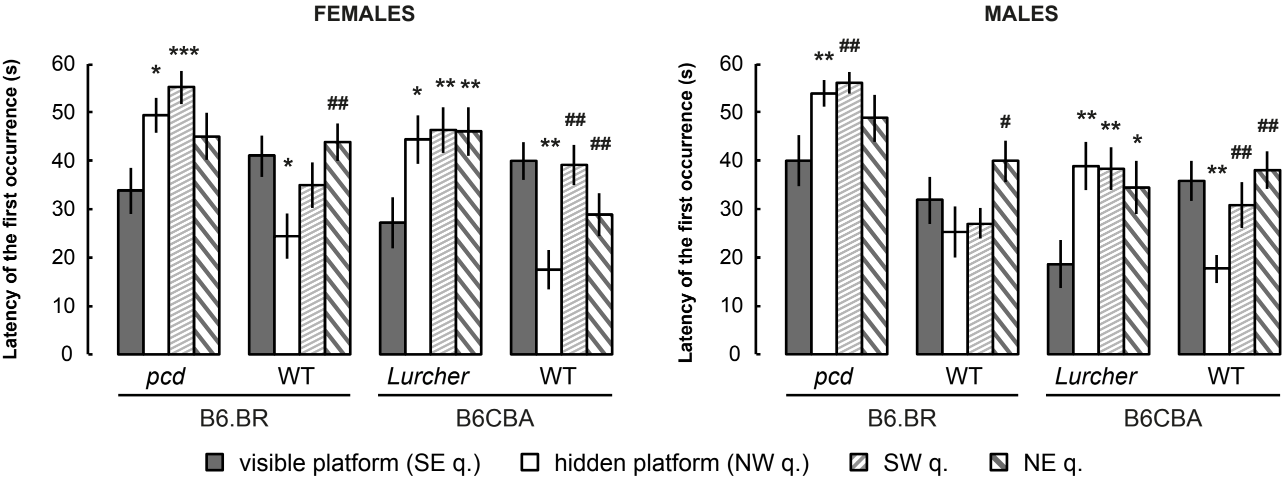

Supplementary Figure 2 - Morris water maze (probe trial): Latency of the first occurrence in the zone of former localization of the visible (center of the SE quadrant) and hidden (center of the NW quadrant) platform in the first trial (E-starting position) of day-session 12. Latencies of the first occurrence in hypothetical platforms localized in the middle of SW and NE quadrants, where the platform has never been placed are also shown. Both B6.BR and B6CBA wild type mice appeared earlier in the zone of previous localization of the hidden platform. On the other hand, cerebellar mutants showed shorter latency of first occurrence for the zone of former localization of the visible platform than for the hidden platform zone. For comparison of individual zones with visible platform zone: * $p < 0.05$, ** $p < 0.01$ and *** $p < 0.001$. For comparison of individual zones with hidden platform zone: # $p < 0.05$ and ## $p < 0.01$. Data are presented as mean ± SEM.
