# Supplementary material for: Mutation-related differences in exploratory, spatial, and depressive-like behavior in pcd and Lurcher cerebellar mutant mice
Source: Front Behav Neurosci. 2015 May 12;9:116. doi: 10.3389/fnbeh.2015.00116 (PMC4429248; doi:10.3389/fnbeh.2015.00116)
Supplement: Supplementary file 3 [file Table1.PDF]

Supplementary Table 1 – Statistical significances of the between-group factors (type, strain and sex) and their interactions for body weight (pooled cohorts 1 and 2) as well as following planned comparisons. Permutational three-way ANOVA with repeated measurements and permutational two sample t-test.

**Three-way ANOVA**		
Between-group factors	$F_{(1,245)}$	p
Type	463.97	< 0.001
Strain	159.43	< 0.001
Sex	450.75	< 0.001
Type:Strain	86.47	< 0.001
Type:Sex	11.88	< 0.001
Strain:Sex	2.33	n.s.
Type:Strain:Sex	0.13	n.s.
**Two sample t-test**		
Planned comparisons	t	p
**Females**		
pcd vs. wild type B6.BR	-20.56	< 0.001
Lurcher vs. wild type B6CBA	-4.85	< 0.001
pcd vs. *Lurcher*	-11.58	< 0.001
WT B6.BR vs. wild type B6CBA	-1.42	n.s.
**Males**		
pcd vs. wild type B6.BR	-12.48	< 0.001
Lurcher vs. wild type B6CBA	-8.16	< 0.001
pcd vs. *Lurcher*	-9.33	< 0.001
WT B6.BR vs. wild type B6CBA	-2.10	0.037
