# Supplementary material for: Mutation-related differences in exploratory, spatial, and depressive-like behavior in pcd and Lurcher cerebellar mutant mice
Source: Front Behav Neurosci. 2015 May 12;9:116. doi: 10.3389/fnbeh.2015.00116 (PMC4429248; doi:10.3389/fnbeh.2015.00116)
Supplement: Supplementary file 4 [file Table2.PDF]

Supplementary Table 2 – Forced swimming test: paired comparison of the first (time-bout 1) and the last (time-bout 3) 5 min intervals for each day-session. Permutational paired t-test.

Groups	Day-session	Females		Males	
		t	*p*	*t*	*p*
pcd B6.BR	1	-3.69	0.005	1.14	n.s.
	2	-3.86	0.004	-1.95	n.s.
	3	-2.22	0.05	-3.26	0.004
wild type B6.BR	1	0.36	n.s.	-2.16	0.045
	2	0.48	n.s.	0.13	n.s.
	3	-1.22	n.s.	0.46	n.s.
Lurcher B6CBA	1	-1.48	0.029	0.82	n.s.
	2	-1.17	n.s.	-1.05	n.s.
	3	-0.37	n.s.	-1.78	n.s.
wild type B6CBA	1	-2.70	0.009	-2.54	0.021
	2	-2.40	0.034	-2.26	0.044
	3	-0.92	n.s.	-1.05	n.s.
